# Transcriptomic Temperature Stress Responses Show Differentiation Between Biomes for Diverse Plants

**DOI:** 10.1093/gbe/evaf056

**Published:** 2025-03-25

**Authors:** Samuel C Andrew, Rosalie J Harris, Chris Coppin, Adrienne B Nicotra, Andrea Leigh, Karel Mokany

**Affiliations:** Agriculture and Food, CSIRO, Canberra, Australian Capital Territory, Australia; Division of Plant Sciences, Research School of Biology, The Australian National University, Canberra, Australian Capital Territory 2600, Australia; Agriculture and Food, CSIRO, Canberra, Australian Capital Territory, Australia; Division of Plant Sciences, Research School of Biology, The Australian National University, Canberra, Australian Capital Territory 2600, Australia; School of Life Sciences, University of Technology Sydney, Broadway, New South Wales, Australia; Agriculture and Food, CSIRO, Canberra, Australian Capital Territory, Australia

**Keywords:** climate change vulnerability, local adaptation, de novo transcriptomics, comparative transcriptomics, photosystem II thermal tolerance

## Abstract

Plants are foundational to terrestrial ecosystems, and because they are sessile, they are particularly reliant on physiological plasticity to respond to weather extremes. However, variation in conserved transcriptomic responses to temperature extremes is not well described across plants from contrasting environments. Beyond molecular responses, photosystem II thermal tolerance traits are widely used to assay plant thermal tolerance. To explore options for improving the prediction of thermal tolerance capacity, we investigated variation in the transcriptomic stress responses of 20 native Australian plant species from varied environments, using de novo transcriptome assemblies and 188 RNA-sequencing libraries. We documented gene expression responses for biological processes, to both hot and cold temperature treatments, that were consistent with conserved transcriptomic stress responses seen in model species. The pathways with the most significant responses were generally related to signaling and stress responses. The magnitude of some responses showed differentiation between the species from contrasting arid, alpine, and temperate biomes. This variation among biomes indicated that postheat exposure, alpine and temperate species had greater shifts in expression than arid species and alpine species had weaker responses to the cold treatment. Changes in the median expression of biological processes were also compared to plasticity in photosystem II heat and cold tolerance traits. Gene expression responses showed some expected relationships with photosystem II thermal tolerance plasticity, but these two response types appeared to be mostly independent. Our findings demonstrate the potential for using variation in conserved transcriptomic traits to characterize the sensitivity of plants from diverse taxa to temperature extremes.

## Graphical Abstract

**Graphical Abstract:**
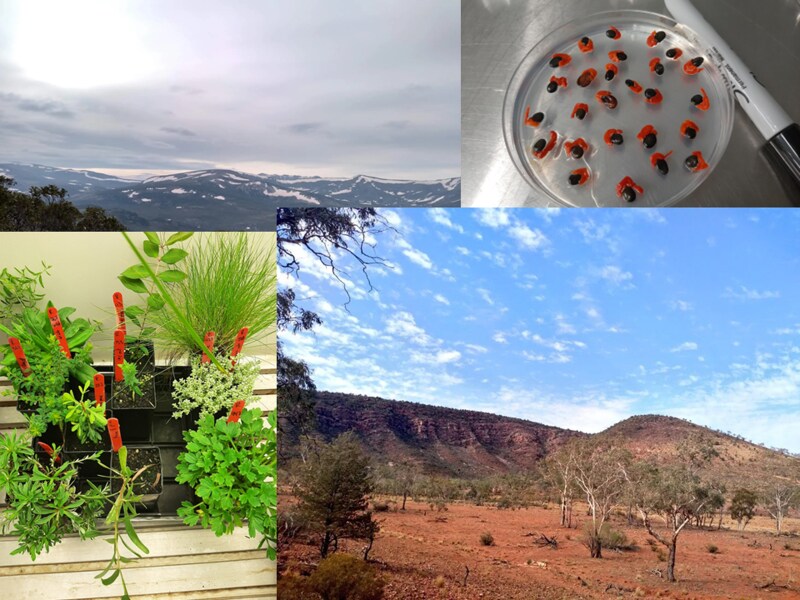

**Significance**

Here, we demonstrate the potential of comparative transcriptomic methods to quantify variation in conserved gene expression responses to temperature stressors, which are understood to be important to thermal tolerance and acclimation. Previously, landscape genomics has focused on genome-wide genetic markers to study signatures of adaptation to climate conditions across species distributions. The assessment of species vulnerability to climate change has also used functional traits, and we believe we have shown here that gene expression traits of the transcriptome have the potential to identify signatures of adaptation to climate. Transcriptomic traits could expand the use of genomic data in conservation biology for plants and potentially other taxonomic groups.

## Introduction

Plants are foundational to terrestrial ecosystems and are increasingly more vulnerable to intensifying temperature extremes (Díaz et al. 2016; Bruelheide et al. 2018; Geange et al. 2021). However, plants are not defenseless when it comes to temperature extremes; underneath an immobile exterior, a whole range of hidden biological responses take place (Wahid et al. 2007; Lancaster and Humphreys 2020; Zhang et al. 2022). Responses to temperature extremes are shared across plants, and it can be expected that these responses would be adapted to local environments (Yeaman et al. 2016; Maher et al. 2018). For plants adapted to different biomes, the optimal strategies for plastic responses to temperature fluctuations could look quite different (Donelson et al. 2023). Variation or diversity in how plants perceive and respond to thermal extremes (Zhu 2016) is likely a critical component for understanding the resilience of plants to tolerate changing climate conditions (Li et al. 2018).

Functional trait diversity (Gallagher et al. 2021) and genetic diversity (Hoban et al. 2023) have been used extensively to inform the assessment of species vulnerability to climate change (Nicotra et al. 2015). However, there is a lack of research to quantify diversity in molecular responses to stress across nonmodel species (DeBiasse and Kelly 2016; Rivera et al. 2021). Molecular stress response traits can be quantified at several genomic levels including the epigenome, transcriptome, proteome, and metabolome (Zhang et al. 2022). With respect to stress responses, the transcriptome is of particular interest because it can rapidly and dynamically respond to environmental stimuli due to evolutionarily conserved signaling cascades (Ghanashyam and Jain 2009; Wilkins et al. 2016; Zhu 2016) and epigenetic regulation (Perrone and Martinelli 2020). The transcriptome should also represent a major force driving changes in the proteome, metabolome, and ultimately cell functioning (Voelckel et al. 2017; Zhang et al. 2022). Genetic variation is critical to regulating heritable variation in the gene expression levels and the responsiveness of gene regulatory networks that allow the plant to respond to stressors. However, linking genetic markers to quantitative traits like molecular stress responses can be difficult (Chaudhary et al. 2020).

Across plants, there are shared transcriptomic responses to temperature stress (Wahid et al. 2007; Nurhasanah Ritonga and Chen 2020). One response to heat stress that is conserved beyond just plants is the upregulation of heat shock proteins (HSPs) and other chaperone proteins that facilitate the folding, aggregation, translocation, and degradation of proteins (Chaudhary et al. 2020). The roles of these proteins become increasingly important as temperatures go beyond the optimal levels for protein folding and subsequently unfolded or misfolded proteins accumulate. This accumulation directly stimulates the increased expression of these chaperone proteins (Feder and Hofmann 1999). The accumulation of reactive oxygen species (ROS) also becomes more problematic as temperatures deviate from optimal levels for metabolism. The accumulation of ROS can cause oxidative stress damage and stimulate other stress responses or even programmed cell death (Mansoor et al. 2022; Mittler et al. 2022). The upregulation of ROS scavenging genes in response to ROS accumulation is also a conserved stress response (Mansoor et al. 2022). A recent meta-analysis of responses to cold stress in six species with paired tolerant and sensitive populations identified a number of conserved responses in controlled environments, including photosynthetic and signaling pathways (Vergata et al. 2022). The ICE-CBF-COR signaling cascade (Hwarari et al. 2022) and stress hormones such as ethylene, gibberellic acid, and jasmonic acid (Wang et al. 2018; Chen et al. 2021; Raza et al. 2021) are also critical for regulating the suppression of growth and the upregulation of cold acclimation genes in response to low temperatures. However, linking variation in these conserved transcriptome responses to variation in individual thermal tolerance has still not been explored in depth, particularly across the diversity of nonmodel plant species (Rivera et al. 2021), but has recently been looked at in a set of 17 *Acacia* congenerics for heat stress responses (Andrew, Simonsen, et al. 2024).

Studies on the thermal tolerance of plants have often focused on the membrane protein complex photosystem II (PSII), because it is highly heat sensitive and the first stage of light-dependent reactions for photosynthesis (Mathur et al. 2014). A number of photosynthetic thermal tolerance (PTT) metrics measure changes in chlorophyll fluorescence with increasing temperature exposure (O'Sullivan et al. 2017; Perez and Feeley 2020; Harris et al. 2024). One such metric is *T*_crit_, which identifies threshold temperatures at which PSII is significantly inhibited, indicated by a sudden rise in basal fluorescence *F*_0_ (Arnold et al. 2021). Measuring PTT provides one method for estimating the capacity of plants to tolerate temperature stress. The quantification of thermal tolerance is made more difficult by PTT plasticity that leads the trait to vary in response to fine-scale environmental conditions (Curtis et al. 2019; Harris et al. 2024), with little known about the molecular basis for this variation.

At the molecular level, mRNA sequencing (RNA-seq) is designed to capture the expression of transcripts for protein-coding genes, and these expression profiles of the transcriptome can change dynamically with environmental stimuli (Imadi et al. 2015). Transcriptome sequencing data are most easily used when aligned to a reference genome, but when such is unavailable, the RNA-seq data can be used for de novo transcriptome assembly to quantify relative transcript abundance (Smith-Unna et al. 2016). This means that with one reasonably affordable sequencing data set, detailed genomic studies of nonmodel organisms can be pursued with both gene sequence and expression data. In contrast to species-specific single nucleotide polymorphism (SNP) markers that are often unique to single-species studies (Ahrens et al. 2018), the conserved sequence of protein-coding genes can be matched across species to more easily compare expression (Rane et al. 2017). Genes that are expected to share a conserved function can be linked across species through several methods. Ortholog genes that are expected to share a common ancestor due to sequence similarity can be used (Rane et al. 2017; Emms and Kelly 2019). Alternatively, gene annotations to related protein families/homologs (Mi et al. 2021) or gene ontology (GO) terms for groups of genes associated with a shared molecular function, cellular component, or biological process (The Gene Ontology Consortium 2019) can be used for comparative analyses.

Transcriptomic responses to standardized temperature treatments could provide an efficient method for capturing variation in how species perceive and respond to temperature stress. Here, we define temperature stress in the broad sense, as being temperature changes that elicit significant biological responses that can be positive (adaptive responses such as acclimation) or negative (costly responses such as reduced reproductive investment or programmed cell death; Rosenfeld et al. 2022). Conserved responses to a stressor should be observable through consistent changes in the expression of genes linked to relevant biological processes (DeBiasse and Kelly 2016). A recent study on 17 Acacia species found clinal variation in transcriptomic stress responses when mapping to a single reference genome for all species (Andrew, Simonsen, et al. 2024). Using de novo transcriptome data, we asked whether variation in relatively conserved responses seen across plant families can be associated with biome of origin and/or thermal tolerance acclimation. By exploring this variation, we also sought to demonstrate the power of comparative transcriptomic methods for assaying variation in the molecular stress responses of diverse nonmodel species. We applied our approach to a suite of 20 native Australian plants (Table 1) that were representative of three contrasting biomes from New South Wales, Australia: arid, alpine, and temperate. We measured changes in gene expression the morning after 3 days of heat stress or cold stress relative to a control group that experienced no change in temperature from benign growing conditions. We expect distantly related species from the same biome to show convergence in the response profile of conserved gene regulatory networks. With arid species expected to show weaker responses to the heat treatment for HSP, ROS, and heat response pathways due to higher tolerance and/or faster recovery. We also expected the responses of alpine species to the cold treatment to be differentiated from arid and temperate species for stress hormone and growth regulation pathways due to their adaptation to cold extremes. Finally, we test for covariation between transcriptomic stress response traits and the PTT acclimation traits we measure.

**Table 1:** Species and de novo transcriptome summary data

Species name	Family	Growth form	Biome	Control *n*	Cold *n*	Hot *n*	Complete BUSCO	Frag BUSCO	Missing BUSCO	Retained transcripts
*Ewartia nubergia*	Asteraceae	Small herb	Alpine	2	2	3	85.8	11.1	3.1	11,900
*Leptorhynchos squamatus*	Asteraceae	Small herb	Alpine	4	4	4	74.3	17.9	7.8	12,361
*Xerochrysum subundulatum*	Asteraceae	Herb	Alpine	4	3	4	75.8	16.9	7.3	12,106
*Oxylobium ellipticum*	Fabaceae	Shrub	Alpine	4	4	4	79.5	17.2	3.3	12,895
*Eucalyptus pauciflora*	Myrtaceae	Tree	Alpine	1	1	2	74.4	18.8	6.8	12,300
*Poa costiniana*	Poaceae	Grass	Alpine	4	3	4	60.7	28.2	11.1	13,192
*Ranunculus graniticola*	Ranunculaceae	Herb	Alpine	4	3	4	77.6	16.7	5.7	10,853
*Capparis mitchelli*	Capparaceae	Tree	Arid	1	1	1	75.5	20.2	4.3	12,843
*Casuarina pauper*	Casuarinaceae	Tree	Arid	1	1	1	63.3	27.1	9.6	12,317
*Acacia aneura*	Fabaceae	Tree	Arid	3	3	3	74.1	18.8	7.1	12,852
*Acacia salicina*	Fabaceae	Tree	Arid	4	4	4	76.7	17.9	5.4	11,405
*Acacia victoriae*	Fabaceae	Shrub/tree	Arid	3	4	4	68.7	24.2	7.1	12,228
*Flindersia maculosa*	Rutaceae	Tree	Arid	2	2	1	82.1	14.1	3.8	11,523
*Dodonaea viscosa*	Sapindaceae	Shrub	Arid	4	4	4	62.4	28	9.6	10,949
*Lomandra longifolia*	Asparagaceae	Herb	Temp	4	3	4	69.9	23.3	6.8	12,767
*Carex appressa*	Cyperaceae	Sedge	Temp	3	4	4	72.2	20.5	7.3	10,572
*Acacia binervata*	Fabaceae	Shrub/tree	Temp	4	4	4	57.4	31.1	11.5	13,293
*Acacia longifolia*	Fabaceae	Shrub/tree	Temp	3	4	4	60.7	27.8	11.5	11,762
*Pittosporum undulatum*	Pittosporaceae	Tree	Temp	3	3	3	88.5	9.9	1.6	11,571
*Banksia integrifolia*	Proteaceae	Tree	Temp	3	4	4	72.4	20.9	6.7	10,177
…	…	…	Average	3.05	3.05	3.3	72.6	20.53	6.87	11,993
…	…	…	Minimum	1	1	1	57.4	9.9	1.6	10,177
…	…	…	Maximum	4	4	4	88.5	31.1	11.5	13,293

The “Control *n*” column has the number of libraries sequenced from control (25 °C day to 15 °C night) growth cabinets. The target was four libraries for each treatment per species, but to fill gaps, some extra species were added at one per treatment. The “Cold *n*” column has the number of libraries sequenced from cold treatment (−2 °C night) growth cabinets. The “Hot *n*” column has the number of libraries sequenced from hot treatment (40 °C day) growth cabinets. The “Complete BUSCO,” “Frag BUSCO,” and “Missing BUSCO” columns have the summary stats from the BUSCO check of *Trinity* de novo transcriptome assemblies. The “Retained transcripts” column has the total number of transcripts retained after filtering for low expression.

## Results

### Transcriptomic Temperature Stress Responses and Interspecies Variation

**Fig. 1.**
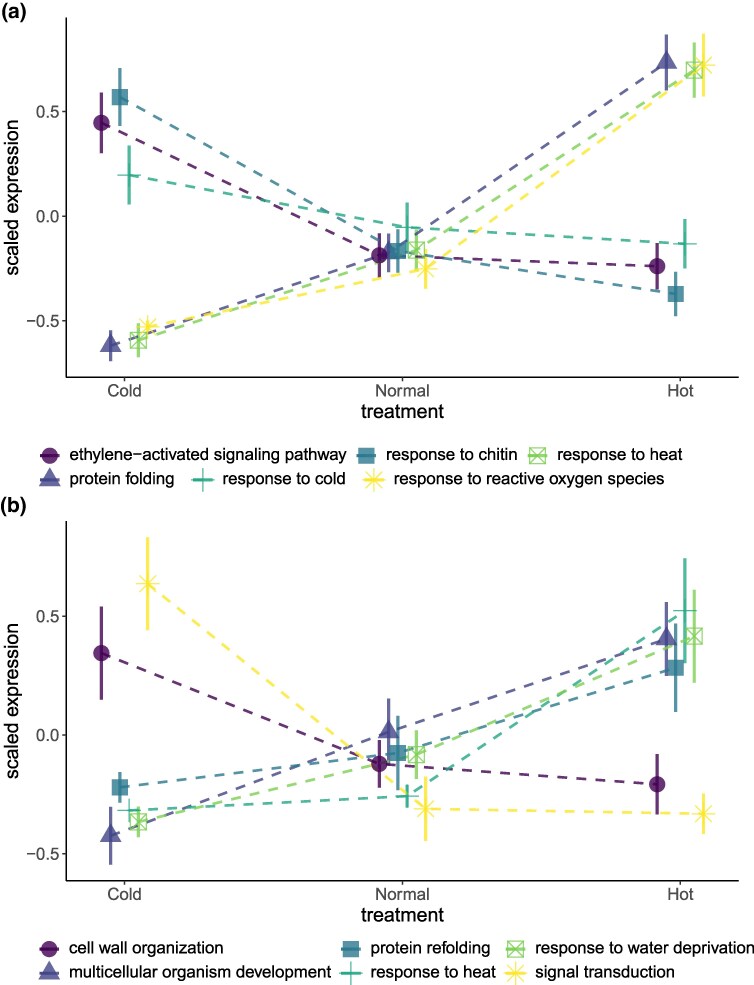
Strongest transcriptomic response to temperature treatments. Tissue samples were taken on the morning after three days of 40 °C heat or three nights of −2 °C cold treatments. a) The boot-strapped median expression values of GO terms. The mean across individuals from 20 species is plotted for each treatment with SE bars (total *n* = 188 individual RNA-seq libraries). See full model results in supplementary table S1, Supplementary Material online. b) Variation in expression for stress response orthogroups. See full model results in supplementary table S2, Supplementary Material online. Expression values were scaled (with mean 0 and SD of 1) prior to calculating means to make trends comparable across GO terms.

The boot-strapped median expression of genes for temperature-related biological process GO terms showed significant and consistent responses across species to both the heat and cold treatments (Fig. 1a; supplementary table S1, Supplementary Material online). From linear mixed models (LMMs) with expression as the response variable, the GO terms most strongly upregulated across the 20 species, post the 3 days of heat treatment (40 °C days), relative to the controls (25 °C days), were protein folding (GO: 0006457, *t*_166_ = 7.48), response to ROS (GO: 0000302, *t*_166_ = 7.05), and response to heat (GO: 0009408, *t*_166_ = 6.25; see full results in supplementary table S1, Supplementary Material online, which includes the top 20 most significant GO terms out of 135 tested). In response to the cold treatment (−2 °C nights relative to 15 °C controls), the cellular response to hypoxia (GO: 0071456, *t*_166_ = 3.70) and response to cold (GO: 0009409, *t*_166_ = 2.43) stress response GO terms were upregulated. However, the GO terms most strongly upregulated after the three nights of cold treatment were response to chitin (GO: 0010200, *t*_166_ = 4.93) and ethylene-activated signaling pathway (GO: 0009873, *t*_166_ = 4.20). Simultaneously, major biological processes such as DNA replication (GO: 0006260, *t*_166_ = −6.55), photosynthesis (GO: 0015979, *t*_166_ = −5.41), and photorespiration (GO: 0009853, *t*_166_ = −4.41) were strongly downregulated after the cold treatment, when species effects are also accounted for.

In the same LMMs comparing median expression levels of individuals across treatments, many GO terms showed strong differentiation in expression between species, according to interclass correlation coefficients (ICCs) that represent the proportion of variance partitioned between species random factor levels (ICC mean = 0.45, min = 0.08, max = 0.81). The variance explained by the fixed effect of treatment was generally less strong (treatment fixed effect marginal *R*^2^ [*mR*^2^] mean = 0.04, min = 0.00, max = 0.31). The response to ROS (*mR*^2^ = 0.29, ICC = 0.09) and response to heat (*mR*^2^ = 0.29, ICC = 0.10) GO terms being two notable exceptions when treatment had a higher explanatory power than species (supplementary table S1, Supplementary Material online).

We identified 78 orthogroups that were expressed in 12 or more of the 20 study species. These orthogroups also displayed trends that were comparable to the median expression of the GO terms with which they were most strongly associated (Fig. 1b; supplementary table S2, Supplementary Material online). In particular, the OG0000018 orthogroup, which included genes with HSP homolog annotations, was significantly upregulated in response to the heat treatment (*t*_120_ = 4.81). Another orthogroup, linked to aquaporin genes that transport water across membranes (OG0000039), was also upregulated in response to the heat treatment (*t*_129_ = 3.76).

**Fig. 2.**
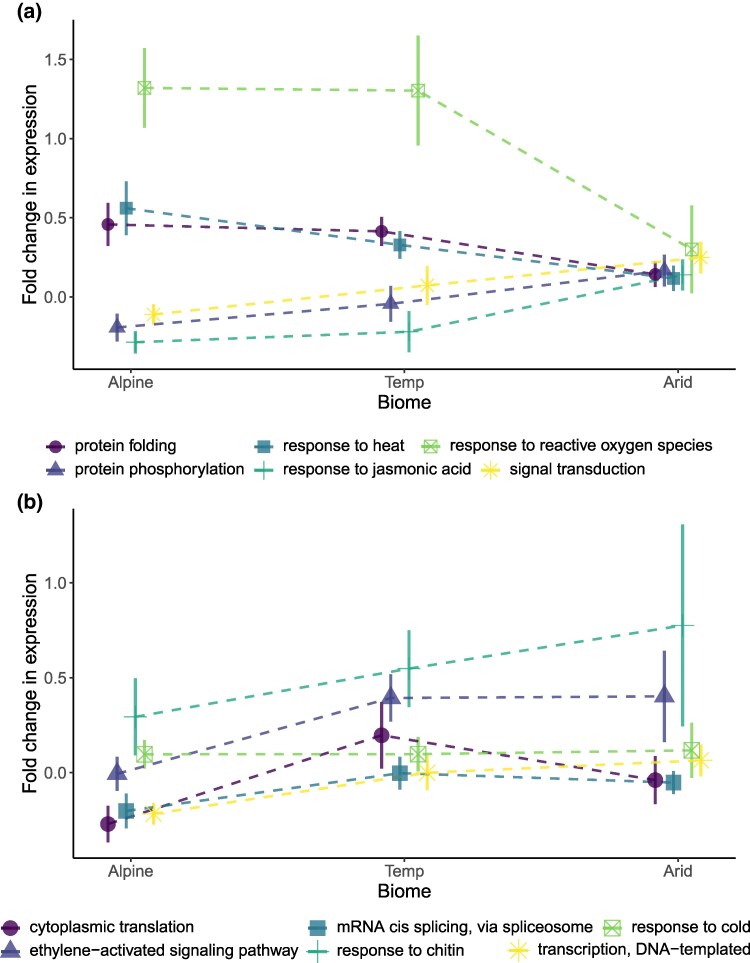
Variation in responses across biomes. a) Responses to the heat treatment that vary between biome. b) Responses to the cold treatment that vary between biome. For each combination of species by treatment, the average boot-strapped expression was used to calculate the FC between treatments. These FC values were *log*2 transformed so decreases in the expression of a biological process in response to stress are shown as negative values and upregulation as positive values. These species by treatment averages were used to calculate the mean and SE values for each combination of treatment and biome. See full model results in supplementary table S3, Supplementary Material online.

We also found some differentiation in the magnitude of responses between the species from different biomes (supplementary table S3, Supplementary Material online; Fig. 2). In general, responses to heat stress were more pronounced in temperate and alpine species when compared to arid species (Fig. 2a). The most relevant GO terms with the strongest differentiation in heat responses were response to jasmonic acid (GO: 0009753, *R*^2^ = 0.40, *P* = 0.014), response to ROS (GO: 0000302, *R*^2^ = 0.33, *P* = 0.034), signal transduction (GO: 0007165, *R*^2^ = 0.30, *P* = 0.47), protein phosphorylation (GO: 0006468, *R*^2^ = 0.29, *P* = 0.056), response to heat (GO: 0009408, *R*^2^ = 0.29, *P* = 0.057), and protein folding (GO: 0006457, *R*^2^ = 0.24, *P* = 0.099). In contrast, for the cold treatment, the GO terms with the strongest differentiation between biomes were GO terms with weaker responses in alpine species (Fig. 2b). The GO terms with the most differentiated for cold responses are transcription, DNA templated (GO: 0006351, *R*^2^ = 0.31, *P* = 0.042), cytoplasmic translation (GO: 0002181, *R*^2^ = 0.26, *P* = 0.074), response to light stimulus (GO: 0009416, *R*^2^ = 0.26, *P* = 0.074), gibberellic acid-mediated signaling pathway (GO: 0009740, *R*^2^ = 0.24, *P* = 0.101), and ethylene-activated signaling pathway (GO: 0009873, *R*^2^ = 0.19, *P* = 0.171).

### PTT Acclimation Associations with Transcriptome Responses

PTT traits also showed signs of acclimation in response to the temperature treatments and variation across species (supplementary fig. S1, Supplementary Material online). The heat tolerance traits of *T*_crit-hot_ and *T*_max-hot_ were significantly higher in response to the heat treatment and, to a lesser extent, in response to the cold treatment (supplementary table S4, Supplementary Material online). When we related *T*_crit-hot_ acclimation (difference between heat and control treatments) to changes in the expression of biological processes, we found that stress signaling pathways had a positive association with acclimation (supplementary fig. S2, Supplementary Material online; Table 2). For example, ethylene-activated signaling pathway (GO: 0009873), plant-type hypersensitive response (GO: 0009626), defense response (GO: 0006952), and cellular response to hypoxia (GO: 0071456) all had significant positive associations with *T*_crit-hot_ acclimation (supplementary fig. S2, Supplementary Material online; Table 2). Similarly, the stress hormone families brassinosteroid-mediated signaling pathway (GO: 0009742) and response to salicylic acid (GO: 0009751) had positive associations with *T*_max-hot_ acclimation (supplementary fig. S2g and h, Supplementary Material online; Table 2).

**Table 2:** Relationships between heat tolerance acclimation and gene expression responses

GO ID	GO term	Trait	Min *n*	Adj. *R*^2^	*t*-value	*P*-value	FC median	FC min	FC max
GO: 0009873	ethylene-activated signaling pathway	Tcrit_hot	26	0.24	2.64	0.0166	0.10	−0.66	0.82
GO: 0009626	plant-type hypersensitive response	Tcrit_hot	16	0.19	2.33	0.0317	0.03	−0.90	0.81
GO: 0030433	ubiquitin-dependent ERAD pathway	Tcrit_hot	11	0.18	2.29	0.0345	0.07	−0.49	0.68
GO: 0006952	defense response	Tcrit_hot	101	0.17	2.19	0.0423	0.01	−0.54	0.48
GO: 0071456	Cellular response to hypoxia	*T*_crit_hot_	25	0.16	2.15	0.0458	0.02	−0.57	0.54
GO: 0009617	Response to bacterium	*T*_crit_hot_	16	0.15	2.07	0.0531	−0.11	−0.77	0.50
GO: 0031146	SCF-dependent proteasomal ubiquitin-dependent protein catabolic process	*T*_crit_hot_	10	0.15	2.11	0.0492	−0.01	−0.53	0.34
GO: 0045087	Innate immune response	*T*_crit_hot_	20	0.15	2.06	0.0540	0.04	−0.56	0.40
GO: 0006468	Protein phosphorylation	*T*_crit_hot_	50	0.14	2.03	0.0578	0.05	−0.58	0.67
GO: 0009723	Response to ethylene	*T*_crit_hot_	18	0.14	2.02	0.0581	−0.01	−0.65	0.68
GO: 0009742	Brassinosteroid-mediated signaling pathway	*T*_max_hot_	11	0.21	2.43	0.0256	0.00	−0.58	0.68
GO: 0000413	Protein peptidyl-prolyl isomerization	*T*_max_hot_	10	0.20	−2.37	0.0293	0.11	−0.23	0.68
GO: 0009617	Response to bacterium	*T*_max_hot_	16	0.15	2.09	0.0512	−0.11	−0.77	0.50
GO: 0009751	Response to salicylic acid	*T*_max_hot_	16	0.13	1.95	0.0675	−0.13	−1.08	0.70
GO: 0009753	Response to jasmonic acid	*T*_max_hot_	14	0.11	1.81	0.0874	−0.19	−0.65	0.56
GO: 0009873	Ethylene-activated signaling pathway	*T*_max_hot_	26	0.11	1.86	0.0791	0.10	−0.66	0.82
GO: 0009651	Response to salt stress	*T*_max_hot_	71	0.09	1.67	0.1129	0.12	−0.24	0.48
GO: 0015979	Photosynthesis	*T*_max_hot_	40	0.08	−1.60	0.1271	−0.03	−0.48	0.64
GO: 0032259	Methylation	*T*_max_hot_	36	0.08	1.64	0.1177	−0.05	−0.24	0.73
GO: 0051085	Chaperone cofactor-dependent protein refolding	*T*_max_hot_	10	0.08	−1.66	0.1142	0.11	−0.55	1.02

Results for linear models are presented for *T*_crit-hot_ and *T*_max-hot_ acclimation. The top ten biological process GO terms that have the strongest relationships with acclimation (highest adjusted *R*^2^ values) are reported for each trait. The “Min *n*” column reports the minimum number of genes used to calculate boot-strapped median expression for species based on the number of genes annotated across species for the difference between hot and control treatments. The “FC median,” “FC min,” and “FC max” report the median, minimum, and maximum FC values across species for each GO term. Results are ordered by trait then by adjusted *R*^2^ values (“Adj. R^2^”) in descending order.

The *T*_crit-cold_ and *T*_max-cold_ acclimation of species ranged between species losing cold tolerance or gaining cold tolerance relative to control plants (supplementary fig. S1, Supplementary Material online). Species that had a worsening cold tolerance performance (i.e. less negative critical temperatures closer to 0 °C) generally had also more strongly downregulated major biological processes in response to the cold treatment (Table 3). For example, chlorophyll biosynthetic process (GO: 0015995) and regulation of gene expression (GO: 0010468) were generally downregulated in species with worsening *T*_crit-cold_ performance; these species also generally had lower upregulation of response to cold (GO: 0009409) (supplementary fig. S3, Supplementary Material online). Consistently, the biological processes of translation (GO: 0006412), photosynthesis (GO: 0015979), and circadian rhythm (GO: 0007623) were generally more strongly downregulated by species that lost *T*_max-cold_ capacity. However, relationships between *T*_max-cold_ acclimation and gene expression responses were weaker and not significant (Table 3).

**Table 3:** Relationships between cold tolerance acclimation and gene expression responses

GO ID	GO term	Trait	Min *n*	Adj. *R*^2^	t-value	*P*-value	FC median	FC min	FC max
GO: 0015995	Chlorophyll biosynthetic process	*T*_crit_cold_	13	0.39	−3.63	0.0019	−0.18	−1.02	0.21
GO: 0009416	Response to light stimulus	*T*_crit_cold_	37	0.25	−2.69	0.0150	−0.17	−0.53	0.34
GO: 0009740	Gibberellic acid-mediated signaling pathway	*T*_crit_cold_	11	0.14	−2.02	0.0580	−0.19	−0.46	0.79
GO: 0009736	Cytokinin-activated signaling pathway	*T*_crit_cold_	10	0.12	−1.88	0.0769	−0.15	−0.75	0.47
GO: 0010468	Regulation of gene expression	*T*_crit_cold_	22	0.12	−1.86	0.0786	−0.11	−0.43	0.57
GO: 0018298	Protein-chromophore linkage	*T*_crit_cold_	14	0.12	−1.89	0.0752	−0.57	−1.92	0.12
GO: 0009845	Seed germination	*T*_crit_cold_	16	0.10	−1.75	0.0975	0.00	−0.53	0.87
GO: 0006412	Translation	*T*_crit_cold_	70	0.09	−1.67	0.1126	−0.14	−0.59	0.72
GO: 0009626	Plant-type hypersensitive response	*T*_crit_cold_	16	0.09	−1.67	0.1117	0.15	−0.53	1.58
GO: 0009742	Brassinosteroid-mediated signaling pathway	*T*_crit_cold_	11	0.09	−1.72	0.1018	0.01	−0.42	0.75
GO: 0015979	Photosynthesis	*T*_max_cold_	40	0.07	−1.53	0.1432	−0.30	−0.84	0.08
GO: 0007623	Circadian rhythm	*T*_max_cold_	29	0.06	−1.50	0.1504	0.07	−0.32	0.51
GO: 0048316	Seed development	*T*_max_cold_	11	0.06	1.47	0.1577	−0.14	−0.34	0.59
GO: 0000413	Protein peptidyl-prolyl isomerization	*T*_max_cold_	10	0.01	−1.05	0.3076	−0.13	−0.47	0.37
GO: 0009965	Leaf morphogenesis	*T*_max_cold_	15	0.01	1.06	0.3025	−0.01	−0.47	0.17
GO: 0006730	One-carbon metabolic process	*T*_max_cold_	10	0.00	−1.03	0.3185	−0.12	−0.67	1.25
GO: 0009624	Response to nematode	*T*_max_cold_	10	0.00	0.98	0.3409	−0.05	−0.59	0.62
GO: 0009846	Pollen germination	*T*_max_cold_	12	0.00	−0.96	0.3514	−0.04	−0.81	0.24
GO: 0015995	Chlorophyll biosynthetic process	*T*_max_cold_	13	0.00	−0.98	0.3379	−0.18	−1.02	0.21
GO: 0018298	Protein-chromophore linkage	*T*_max_cold_	14	0.00	−0.96	0.3479	−0.57	−1.92	0.12

Results for linear models are presented for *T*_crit-cold_ and *T*_max-cold_ acclimation. The top ten biological process GO terms that have the strongest relationships with acclimation (highest adjusted *R*^2^ values) are reported for each trait. The “Min *n*” column reports the minimum number of genes used to calculate boot-strapped median expression for species based on the number of genes annotated across species for each GO term. The “FC median,” “FC min,” and “FC max” report the median, minimum and maximum FC values across species for the difference between cold and control treatments. Results are ordered by trait then by adjusted *R*^2^ values (“Adj. *R*^2^”) in descending order.

## Discussion

The responses of plants to extreme or stressful changes in temperature can be considered as adaptive plasticity if these responses are beneficial to performance and fitness (Nicotra et al. 2010; Donelson et al. 2023). We define temperature stress responses as significant biological responses to deviations from normal temperatures (Rosenfeld et al. 2022). Conserved molecular stress responses to extreme temperatures have been well studied in model plant species (Zhu 2016; Zhang et al. 2022), but variation in these responses has not been linked to thermal tolerance capacity for nonmodel species (Ding et al. 2019; Chaudhary et al. 2020). Using de novo transcriptome assemblies to calculate the boot-strapped median expression of GO terms and mean expression of orthogroups, we document consistent changes in gene expression across the challenging hot and cold treatments for our 20 species (Fig. 1). After accounting for differentiation in expression between species, we found the GO terms most strongly impacted by temperature treatments to be also linked with known transcriptomic temperature stress responses (Fig. 1; supplementary tables S1 and S2, Supplementary Material online). In some cases when the direction of the response to temperature was consistent, the magnitude of the response also varied consistently between species from different biomes (Fig. 2). The differences between the two extreme biomes, observed the morning after 3 days of exposure, can be summarized as weaker responses to heat in arid species (Fig. 2a) and weaker responses to cold in alpine species (Fig. 2b). We expect similar stress responses from distantly related species from the same biomes due to convergent selection on genetic markers that influence variation in gene regulatory networks. Below, we highlight the value of comparing transcriptomic responses across diverse species at the more general level of biological processes and orthogroups.

### Linking Comparative Transcriptomics to Functional Trait Ecology

The possibility of using variation in transcriptomic stress responses to assess thermal tolerance and climate change vulnerability extends from the use of functional traits to predict the fundamental environmental niche of species (McGill et al. 2006; Andrew et al. 2022). Plant functional traits can characterize plant life history strategies (Díaz et al. 2016) and be used to explain the distribution of ecological variation (Westoby 1998; Westoby et al. 2002). Robust relationships between climate metrics and the distribution of functional trait variation establish the importance of these traits to climate adaptation (Wright et al. 2017, 2004; Laughlin et al. 2021). Additionally, combinations of functional traits can have even stronger relationships with species-realized climate limits compared to individual traits (Andrew et al. 2022). Functional trait diversity can still be high within plant communities, but this diversity also shows signs of environmental filtering (Bruelheide et al. 2018; Andrew et al. 2021). Molecular responses to stress provide an opportunity to add important detail to our description of plant adaptive strategies (Razgour et al. 2018; Hoffmann et al. 2021).

For comparative transcriptomic analyses such as ours, which include species from diverse families and genera, it is not possible to map transcript sequences to a single reference genome or to identify one-to-one ortholog genes across species. Using coarser resolution comparisons with suites of biological processes, we were able to confirm responses to temperature treatments that are consistent to those seen in studies of model species. The clearest responses to the heat treatment include the GO terms response to heat, protein folding, and response to ROS that were upregulated after the 3-day heat treatment, and all groups saw an average increase in median expression of about 1 SD (Fig. 1a). There were also other conserved responses to the heat treatment that were characteristic of a high-temperature stress response (supplementary table S1, Supplementary Material online). These include DNA repair (GO: 0006281, *t*_166_ = 3.47), chloroplast organization (GO: 0009658, *t*_166_ = 4.20), and mRNA processing (GO: 0006397, *t*_166_ = 4.67). These results demonstrate how the boot-strapped median expression of GO terms can capture responses to temperature that have been seen in studies of individual species.

The response to cold GO term was generally upregulated in the cold treatment, but not to the same extent as the main heat treatment responses. However, the response to cold change is potentially still notable when considering that most biological processes had lower median expression levels after the cold treatment (77% of GO terms with lower median expression across species). Two of the most strongly upregulated GO terms in response to the cold treatment were the response to chitin and ethylene-activated signaling pathway (supplementary table S1, Supplementary Material online). The ethylene pathway and other stress hormone pathways could be regulating the suppression of other biological processes (Chen et al. 2022). The response to chitin pathway is also linked to regulating stress responses to pathogens and a range of environmental stressors (Vaghela et al. 2022). These pathways and other stress hormone pathways that suppress growth are stimulated by a number of stressors to limit energy expenditure and ROS accumulation during stress (Zhang et al. 2022). The regulation of cold acclimation is thought to be led by the efficiency of the ICE transcription factors in the ICE-CBF-COR signaling cascade, and these transcription factors have improved efficiency when phosphorylated by calcium-sensitive kinase genes (Hwarari et al. 2022). Interestingly, two orthogroups linked to signal transduction were upregulated in response to the cold treatment (OG0000242 and OG0000020; supplementary table S2, Supplementary Material online). Both these orthogroups mainly include homologs of CBL-interacting protein kinase genes that are expected to activated by calcium inflows to the cell in response to cold and other stressors (Zhang et al. 2022) to propagate signaling cascades that regulate gene expression (Mo et al. 2018). Variation in conserved cold-sensitive signaling cascades could be critical for describing cold sensitivity and acclimation capacity.

Differentiation between the study species from the three contrasting biomes in our study is most clearly seen in stress-related GO terms (supplementary table S3, Supplementary Material online). The species with the strongest change in expression after 3 days of the heat treatment were from the temperate and alpine biomes (Fig. 2a). This result highlights the importance of understanding when a large change in gene expression indicates positive fitness-increasing plasticity versus a signal of a more chronic or negative stress or just a slower recovery rate. For this study, samples were taken at the same time of morning for both the cold and heat treatments after the third day of exposure; for the heat treatment, this meant a longer recovery over the third night relative to the cold treatment that had just returned to normal temperatures. The weaker upregulation for protein folding, response to heat, and response to ROS genes in arid species could indicate that they were faster to recover than species native to alpine or temperate regions. Interestingly, the response to jasmonic acid GO term had slightly upregulated levels for arid species and lower levels for alpine and temperate species (Fig. 1a). This result could reflect that variation in jasmonic acid levels can regulate the response to elevated ROS levels (Wang et al. 2020). This result suggests more rapid ROS scavenging in arid zone species resulting in faster recovery and the subsequent suppression of the ROS response through the jasmonic acid pathway at sampling. Responses to unfolded proteins could also show similar recovery patterns. A study of four *Eucalyptus* species found that two species with smaller distributions had higher leaf damage and HSP upregulation due to a heatwave treatment when compared to two species with broad distributions (Aspinwall et al. 2019). Similarly, we found a weaker upregulation of protein-folding genes in arid species (Fig. 2a).

Gene expression data from a single species can appear noisy at the best of times, and we might expect that comparing responses across distantly related species would not be conducive to any overall insights. However, the relationships we find here between transcriptomic responses with both biome of origin and thermal tolerance acclimation suggest important potential for comparative transcriptomics to grow our understanding of species plasticity in response to environmental extremes. A multienvironment trial on a suite of plants with contrasting transcriptomic responses to temperature stressors/stimuli, grown across a strong climate gradient, could be a valuable next step. Performance across sites could help determine the meaning of variation in these responses so traits of the transcriptome can be applied more broadly with other data types to the study of species resilience to climate change.

In contrast to landscape genomic methods that use genome-wide SNP markers to look for outlier loci that show signatures of selection through changes in allele frequency across the distribution of a single species (Ahrens et al. 2018), the current method compares the transcriptome responses of different species that originate from different environments. We expect species from similar environments to converge on similar response strategies through the adaptation of gene regulator networks that control a multifaceted response and are evolutionarily conserved across species (Chen et al. 2022). Species with outlier response patterns could be expected to be vulnerable while for the application of landscape genomic methods, study species that lack outlier loci, genetic diversity, or a capacity to change allele frequencies with climate change could be expected to be vulnerable.

### Linking Transcriptomic Stress Responses to Thermal Tolerance Acclimation

In addition to variation across biomes, we found a range of gene expression responses to be associated with thermal tolerance differences (degree change in PTT between treatments; Tables 2 and 3). Our approach aimed to compare the magnitude of responses to temperature treatments so that covariation between transcriptomic stress responses and PTT acclimation can help develop methods for studying adaptive plasticity (Donelson et al. 2023).

In response to the cold treatment, most GO terms were downregulated. It would be expected that many biological processes in plants would slow down in response to long periods of very low temperatures, and we would see the lag of this effect the morning after exposure. This slowdown could be regulated by the stimulation of stress signaling pathways (Chen et al. 2022). In our study, the species with the greatest reduction in cold tolerance performance (i.e. higher PSII cold tolerance threshold temperatures under stress) also had the strongest downregulation of biological processes under stress (Table 3). Some of the species with the largest loss in *T*_crit-cold_ tolerance were actually alpine species from climates with the most frequent and intense cold exposure, but again the pattern was not uniform (supplementary fig. S3a, Supplementary Material online).

In response to the heat treatment, the species with the highest upregulation of protein folding genes had smaller changes in *T*_max-hot_ (supplementary fig. S2e, Supplementary Material online). This result is consistent with previous findings in *Acacia* where PSII acclimation had a negative relationship with chaperone protein upregulation (Andrew, Simonsen, et al. 2024). Additionally, the species with high *T*_max-hot_ acclimation also had higher upregulation of brassinosteroid and salicylic acid phytohormone pathways (Table 2). These plant hormones are linked to the signaling pathways that regulate stress responses and growth (Chen et al. 2022). Responses for several other stress-related biological processes also had positive associations with *T*_crit-hot_ acclimation (see results in Table 2). These gene expression responses could be helping to stimulate PSII acclimation, but they could also indicate damage accumulation or the downregulation of growth that is associated with changes in PTT (Zhang et al. 2022). The ethylene response pathway was also associated with PTT in an alpine herb *Wahlenbergia ceracea* (Notarnicola et al. 2021). Taken together, high heat tolerance acclimation could be a signal that plants are being more easily pushed beyond their limits and not an indication that high physiological plasticity equals high climate change resilience.

In general, thermal tolerance acclimation to either heat or cold did not seem to be related to phylogeny (supplementary fig. S4, Supplementary Material online), although we have not formally tested this observation here. An example of low phylogenetic signal is *Acacia* species that had a range of rankings for heat acclimation, even when we might expect them all to be highly ranked due to a previous study showing high PSII heat tolerance acclimation in these taxa (Andrew et al. 2023). Variation between studies could be due to diurnal variation in PTT, similar to wheat crops that show lower critical temperatures at sunrise (Posch et al. 2022). Nonetheless, the question of phylogenetic differentiation would be worth exploring in a larger study with a more representative sampling of taxa.

## Conclusion

It stands to reason that if heatwaves become too intense in species' current home range, there is still no guarantee that plants can successfully disperse to cooler neighboring climates due to the potential of cold and/or frost exposure being too high. Based on crop growth models using future climate projections, the frequency of heatwaves is expected to increase without a reduction in frost pressure across large areas of the southern half of inland Australia (Zheng et al. 2015; Hammer et al. 2020). This highlights the benefit of our study in characterizing variation in response to both heat and cold exposure. In general, we demonstrate this point by using transcriptomics to show that species from arid climates have unique responses to heat while species from alpine climates have different responses to cold (Fig. 2; supplementary table S3, Supplementary Material online).

Here, we apply our comparative transcriptomic approach to assess variation in the response of plants to thermal stress. However, the same approach using de novo transcriptome assemblies and the median boot-strapped expression of GO term annotations or orthogroups could be applied to study responses to any stressor in other taxonomic groups. If responses to a stressor are conserved across related species, then the magnitude of the response could show variation that is related to adaptation. Here, we have demonstrated variation in the responses of plants from contrasting biomes that was consistent with our expectations for adaptation to these environments. The adaptive explanation of this variation is yet unclear, for example, when a larger response is a positive indication of tolerance or a negative indicator of damage accumulation. We have only discussed a small proportion of the biological processes with significant trends, but we believe these highlighted groups and others could be used in the future to create transcriptomic metrics of thermal sensitivity in plants. However, data from a broader panel of species being trialed across varied environments are required to elucidate the meaning of these trends. Currently, functional trait data can be sourced for thousands of species to help evaluate adaptive strategies and climate change vulnerability (Butt and Gallagher 2018; Gallagher et al. 2020). Transcriptomic data sets are also being collected for many species (One Thousand Plant Transcriptomes Initiative 2019) but these data do not capture response to standardized environmental stimuli. Variation in transcriptomic responses to benchmark stress treatments could provide valuable functional traits of the transcriptome for predicting the vulnerability of species to future climates (DeBiasse and Kelly 2016).

## Materials and Methods

### Study Design and Sampling

The 20 study species were selected to be representative of the major families and growth forms from three ecosystems that represent the three contrasting biomes of arid, alpine, and coastal temperate environments in Australia (Harris et al. 2024). The arid biome represents an extreme environment that is known for its high maximum temperatures and aridity but will also experience regular frost events. The alpine biome represents vegetation above the treeline in the Australian Alps that experiences the most extreme frost events and the only study community adapted to snow, but the exposed slopes of mountains can also experience high temperatures during summer. The coastal temperate environment represents a more stable climate, with lower temperature fluctuations within a day or across a year and a very low chance of frost. The 20 study species spanned 13 families and 16 genera (Table 1); see Harris et al. (2024) for further details about species selection.

Seeds for the study species were obtained from conservation seed banks, including the Australian National Botanic Gardens Seed Bank and the Australian Botanic Gardens Plant Bank. A single accession of seed was used for germinating each species, and these accessions were collected within a 50-km radius of a target site for each biome, or seedlings with comparable provenance were purchased (Harris et al. 2024). Seedlings were potted in Australian native potting mix and grown under common conditions in a glasshouse at 25 °C day/15 °C night cycles for 3 to 5 months, depending on germination time. Plants were watered daily and fertilized every 2 weeks with low-phosphorus liquid fertilizer.

Plants were then moved to the Australian Plant Phenomics Facility at CSIRO Black Mountain laboratories, where they were kept in Conviron growth chambers for an acclimatization period of 1 to 2 days prior to temperature treatments. Temperature treatments were run for 3 days with the heat treatment being 40 °C days to 15 °C nights and the cold treatment being 25 °C days to −2 °C nights, relative to a control group that experienced no change in temperature from benign growing conditions (25 °C day to 15 °C night). On the morning after the third day, leaf samples were taken between 9:00 AM and 10:30 AM for RNA extraction (see below) and PTT measurements. Assays of thermal tolerance were conducted between 10:00 AM and noon on Day 3 of the experiment when temperatures were between 15 °C and 21 °C in all chambers (see Harris et al. 2024). In brief, for five plants from each species by treatment combination, leaf discs (1 cm^2^) were punched from one leaf per plant and placed into pill boxes moistened with florist foam to maintain turgor. Two Maxi Pulse Amplitude Modulating (PAM) systems (Heinz Walz GmbH, Effeltrich, Germany) were set up, one for *T*_crit-hot_ and one for *T*_crit-cold_ measurements. Each PAM was placed above a Peltier plate (CP-121HT; TE-Technology, Inc., Michigan, USA) regulated by a temperature ramp controller (TC-36–25; TE-Technology, Inc.) and powered by a fixed-voltage power supply (PS-24–13; TE-Technology, Inc.). Cooling rates were set to 15 °C h^−1^ from 20 °C to −25 °C and heating rates to 30 °C h^−1^ from 20 °C to 65 °C (see Arnold et al. 2021 for PAM setup and parametrizations). Leaf discs were randomized on a 48-cell paper array, and a type-T thermocouple (Omega Engineering, USA) was attached to the abaxial side of each leaf, monitored with a 48-channel data Taker DT85 (Lontek, Australia) logging every 5 s. The critical temperatures during heating and cooling, *T*_crit-hot_ and *T*_crit-cold_, were defined as the breakpoint between the slow and fast-rise phases of basal fluorescence, which is an indicator of stress within the thylakoid. Similarly, the maximum and minimum temperatures were extracted, *T*_max-hot_ and T_max-cold_, respectively; all extractions followed protocols from Arnold et al. (2021). The critical temperature values were determined using the *Segmented* package in R. The code we used for this analysis is available at https://github.com/pieterarnold/Tcrit-extraction.

### RNA Extraction and RNA-seq Library Prep

For total RNA extraction from leaf tissue, homogenization was done by grinding samples with liquid nitrogen in a mortar and pestle. Then, the NucleoSpin RNA Plant and Fungi Kit (Macherey-Nagel, Germany) was used for RNA extraction. The standard kit protocol was used except for an adjustment to the lysis buffer as suggested by Ishihara et al. (2016). The lysis buffer aliquot per sample included 400-μl PFL and 50-μl PFR buffers from the NucleoSpin Kit, 100-μl Fruit-mate for RNA Purification (Takara, Japan), and 5-μl ß-mercaptoethanol.

After mRNA isolation with Oligo d(T)_25_ Magnetic Beads (New England BioLabs, Australia), strand-specific RNA-seq libraries were prepared using an in-house template switching protocol. The detailed protocol for library preps is described in Paten et al. (2022). Briefly, two plates of 96 libraries were prepared using custom barcodes. Samples from the different treatments and species were split across the two pools. Samples were sequenced on a single NovaSeq S2 flowcell (300 cycles, 2 × 150 bp) using a lane splitter kit to split the two sample pools onto one lane each. Sequencing was done at Biomolecular Resource Facility at John Curtin School of Medical Research at The Australian National University. The original sequence data for the 192 RNA-seq libraries are published online (Andrew and Mokany 2024). For the 192 libraries, the target was four libraries for each treatment by species combination (Table 1), but to fill gaps, some extra species were added at one library per treatment. We believe the addition of species is still valuable to a comparative analysis of this kind even if sample size is low.

### De Novo Transcriptome Assemblies and Transcript Expression Quantification

Quality assessment and filtering of reads were done with the *fastp* program using default settings (Chen et al. 2018). The number of paired-end reads returned per library prefiltering varied from 7.15 to 44.39 million (mean = 23.76 million) and on average 98% of reads were retained after filtering. For each species, an RNA-seq library from the heat treatment with a high number of reads was selected for de novo transcriptome assembly with *Trinity* (version 2.11.0) and associated packages (Haas et al. 2013). A single individual was used per assembly to prevent genetic variation causing issues for transcript assembly when merging data from multiple individuals. Prior to the *Trinity* assembly, the paired-end reads were further trimmed using *Trimmomatic* (Bolger et al. 2014) using the following settings “HEADCROP:13 LEADING:3 TRAILING:3 SLIDINGWINDOW:4:15 MINLEN:50”. This step helps remove base calls at the start and the end of the reads that have a higher probability of being errors and, therefore, highly detrimental to the assembly process.

*Trinity* assemblies were assessed with BUSCO (version 5.1.2), using the “viridiplantae_odb10” reference and standard settings to check assemble completeness (see results in Table 1). The assembled transcripts were annotated using *TransDecoder* (version 5.5.0) with *Trinotate* (version 3.2.1; Bryant et al. 2017). With *Trinotate*, the sequences were used to search for transcript functional annotations using *blastp*, *blastx*, *domtbl*, and *signapl.* The de novo assemblies and annotations are published online and made publicly available (Andrew, Coppin, et al. 2024).

To quantify gene expression, the RNA-seq libraries for each species were aligned to the de novo assembly for the species using *RSEM* (version 1.3.3). After quantifying gene expression, four libraries (“20_H1,” “15_H1,” “24_C3,” and “21_C2”) were dropped due to low mapping rates that were below 50%. For the remaining libraries, mapping rates ranged from 58.2% to 84.5%.

For all 20 species, the amino acid sequences of the assembled transcripts (.pep files output by *TransDecoder*) were used in an *OrthoFinder* (version 2.5.4) analysis to identify groups of ortholog genes (or “orthogroups”) across species (Emms and Kelly 2019). These groups of genes, which are expected to be related by descent based on sequence similarity across species, were used to compare responses to temperature stimulus across species.

### Data Analysis

All postexpression quantification statistical analyses and figure generation were done in R version 4.2.1 (R Core Team 2016). All R codes for our main analyses are published with the Supplementary material in an online repository (Andrew and Mokany 2024).

The *RSEM* gene expression outputs were standardized across libraries using the average number of reads mapping to a transcript per million reads sequenced (transcripts per million [TPM]). The transcripts (hereafter referred to as “genes”) with an average TPM expression level ≥ 10 were retained for each species. Across the 20 species, the number of retained genes ranged between 10,177 and 13,293 with mean ± SD = 11,993 ± 886. This filtering focused the analysis on a consistent number of genes per species and away from genes with low expression levels or low mapping rates due to poor sequence assembly. For retained genes, the mean TPM expression level was calculated for each species by treatment combination so that differences in expression between treatments could be assessed. The log_2_-transformed fold change (FC) in expression was calculated, with the expression levels of the heat treatment being divided by neutral temperature controls (HvsN) and the cold treatment divided by neutral temperature controls (CvsN), prior to log_2_ transformation.

The annotation report files from *Trinotate* were used to match genes to GO (GO term) annotations. The GO system is a hierarchically classification of genes based on sequence similarity with genes linked to a molecular function, cellular component, or biological process (The Gene Ontology Consortium 2019). The number of genes linked to each GO term was calculated for each species. Genes can have multiple GO terms assigned to from multiple classification levels for each of the three categories. Biological process GO terms with a minimum of ten annotated genes across all species were retained for calculating GO term expression levels. This filtering focused our analysis on 134 biological processes with a range of general to specific functional descriptions. To compare the expression levels of biological processes across species, the boot-strapped median TPM expression levels and FC values were calculated for GO terms. The boot-strapping method used 1,000 resampling subsets of genes linked to each GO term per species. The size of random subsets for each GO term was set to 75% of the minimum number of annotated genes across all species, rounded to the nearest whole integer. The 1,000 resampled median values were then averaged. These boot-strapped median values can be used to assess whether the expression of genes linked to a GO term is going up or down and the magnitude of this change. This method also controls for the number of expressed and annotated genes per species. Boot-strapped median values were calculated for TPM expression per individual library and for FC values at the species level.

In addition to GO terms, there were 220 orthogroups that were present across 15 or more species. The mean expression of genes from each orthogroup was calculated for all individuals from each species. The mean was used due to some orthogroups not having more than one gene per species. After filtering out genes with <10 TPM, only 78 orthogroups that were expressed in 12 or more species were retained for further analyses. We therefore focus the presentation of results on GO terms due to the higher statistical power.

We expect these functional groupings of genes to show conserved responses to temperature treatments that are expected to invoke stress responses. To explore variation in median GO term and mean orthogroup, expression across treatments the *lme4* package (Bates et al. 2015) was used to fit LMMs with treatment as a categorical fixed effect and species ID as a random factor with independent intercepts per level. The mean and SE of expression levels within treatments for all species were also calculated to plot significant trends from LMMs. We also expected variation in the magnitude of these stress responses to vary between species adapted to the contrasting arid, alpine, and temperate biomes. To compare variation in species-level FC values across biomes, linear models were used with biome set as a categorical fixed effect. The species-level FC values for comparisons between the hot versus neutral treatments and cold versus neutral treatments were both assessed.

Finally, we related these gene expression responses to the PTT plasticity of plants. The *T*_crit-hot_ and *T*_max-hot_ acclimation responses were calculated as the difference between the hot and neutral treatments, and *T*_crit-cold_ and *T*_max-cold_ acclimation was the difference between the cold and neutral treatments. We use the term acclimation to refer to the difference between the treatment and control group that could also be thought of as a metric of PTT plasticity and, for this study, does not always indicate an improvement in PTT under stress. These acclimation responses were compared to the corresponding median gene expression FC values using linear models. The FC response of biological processes was used as a continuous predictor to explain variation in photosynthetic acclimation. Due to the relatively low statistical power with only 20 species, we focused on the functional annotations and adjusted *R*^2^ values of the strongest relationships rather than corrected *P*-values.

## Supplementary Material

evaf056_Supplementary_Data

## Data Availability

As agreed with the funders of this research, raw sequence data are made publicly with the RNA-seq library metadata; processed gene expression data, gene annotations, and additional data for analyses are also made available with all Rmarkdown scripts on CSIRO's Data Access Portal (https://doi.org/10.25919/0jj0-cz83). The de novo transcriptome assemblies along with metadata are also made available on CSIRO's Data Access Portal (https://doi.org/10.25919/6mq7-ya76).
