# Supplementary material for: Transcriptomic Temperature Stress Responses Show Differentiation Between Biomes for Diverse Plants
Source: Genome Biol Evol. 2025 Mar 25;17(4):evaf056. doi: 10.1093/gbe/evaf056 (PMC11997244; doi:10.1093/gbe/evaf056)
Supplement: evaf056_Supplementary_Data [file evaf056_supplementary_data.pdf]

Supplementary Results

Transcriptomic temperature stress responses show differentiation between biomes for diverse plants

Samuel C. Andrew, Rosalie J. Harris, Chris Coppin, Adrienne B. Nicotra, Andrea Leigh & Karel Mokany

Supplementary Figures

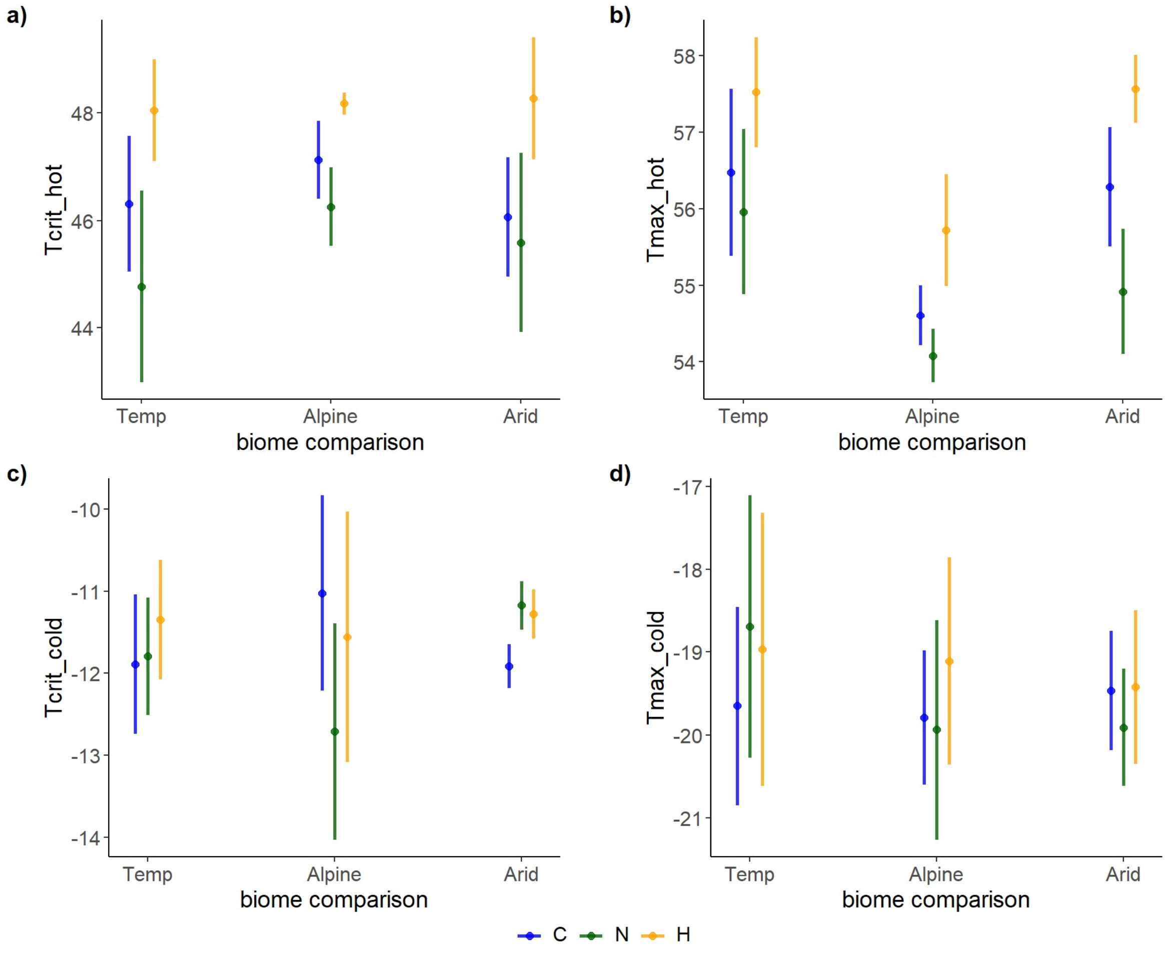

Figure S1. Thermal tolerance measurements across treatment and biome combinations. the mean of each combination is plotted with s.e. bars for variation between species. Only $T_{crit\text{-}hot}$ and $T_{max\text{-}hot}$ show signs of significant improvement under stress (Table S4).

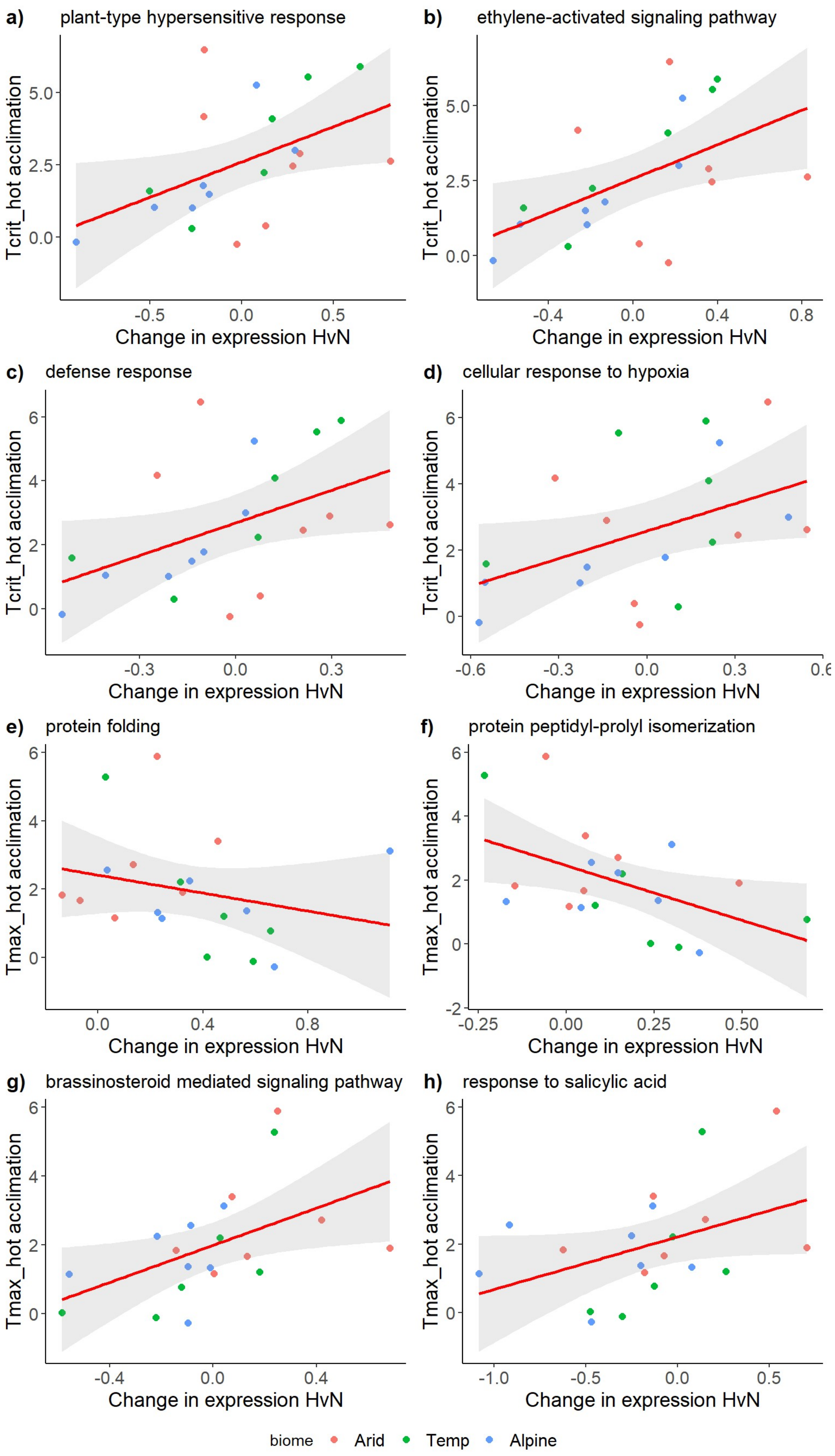

a) plant-type hypersensitive response
b) ethylene-activated signaling pathway
c) defense response
d) cellular response to hypoxia
e) protein folding
f) protein peptidyl-prolyl isomerization
g) brassinosteroid mediated signaling pathway
h) response to salicylic acid
Tcrit_hot acclimation
Tmax_hot acclimation
Change in expression HvN
biome Arid Temp Alpine

Figure S2. Associations between heat tolerance acclimation and changes in expression. Heat tolerance acclimation was calculated as the change in photosynthetic heat tolerance between the hot and control treatments, with positive values indicating improved tolerance under stress. We compare acclimation responses to changes in expression between the hot and control treatments, fold change values are $log2$ transformed. Plots include interesting relationships from an analysis of all GO terms (Table 2). For $T_{crit\text{-}hot}$ examples see panel a) to d) and for $T_{max\text{-}hot}$ examples see e) to h).

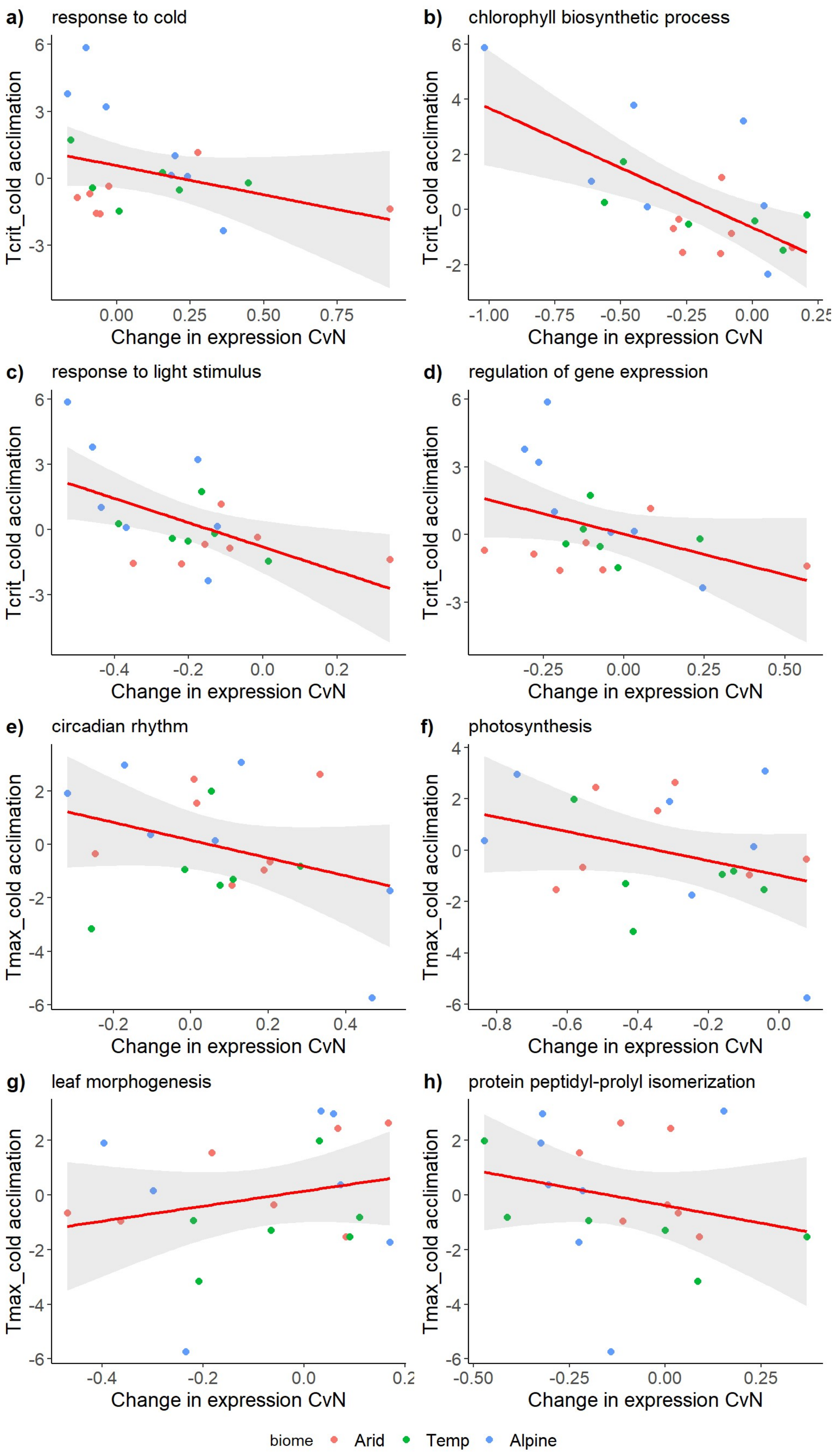

a)
response to cold
Tcrit_cold acclimation
Change in expression CvN
b)
chlorophyll biosynthetic process
Tcrit_cold acclimation
Change in expression CvN
c)
response to light stimulus
Tcrit_cold acclimation
Change in expression CvN
d)
regulation of gene expression
Tcrit_cold acclimation
Change in expression CvN
e)
circadian rhythm
Tmax_cold acclimation
Change in expression CvN
f)
photosynthesis
Tmax_cold acclimation
Change in expression CvN
g)
leaf morphogenesis
Tmax_cold acclimation
Change in expression CvN
h)
protein peptidyl-prolyl isomerization
Tmax_cold acclimation
Change in expression CvN
biome
Arid
Temp
Alpine

Figure S3. Associations between cold tolerance acclimation and changes in expression. Cold tolerance acclimation was calculated as the change in photosynthetic cold tolerance between the cold and control treatments, with negative values indicating improved tolerance under stress. We compare acclimation responses to changes in expression between the cold and control treatments, fold change values are *log*2 transformed. Plots include interesting relationships from an analysis of all GO terms (Table 3). For $T_{crit\text{-}cold}$ examples see panel a) to d) and for $T_{max\text{-}cold}$ examples see e) to h).

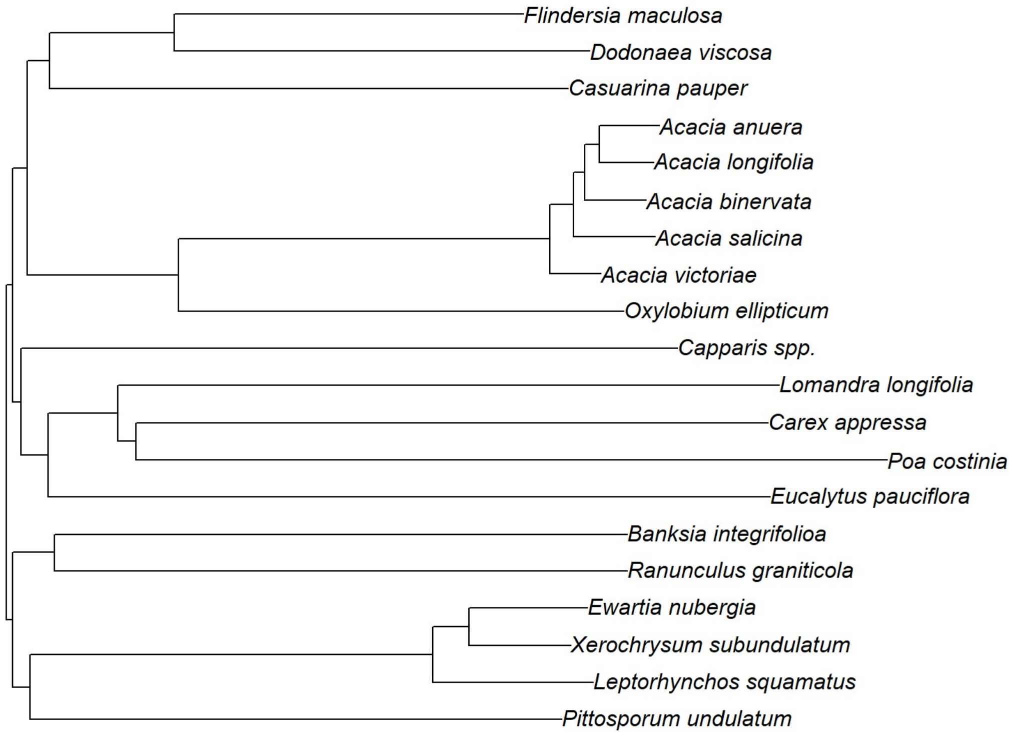

Figure S4. Species tree. The tree was generated from the *OrthFinder* analysis used to identify orthogroups from the *Trinity de novo* transcriptome assemblies.

Supplementary Tables

Table S1. Model results comparing treatments. Using boot-strapped median expression levels for GO terms (gene ontology terms), calculated per plant. The “Min n” column shows the minimum number of transcripts used to calculate median boot-strapped expression per GO term. The marginal R^2 (“mR2”) represents the proportion of variance explained by the fixed effect of treatment. The conditional R^2 (“cR2”) represents the proportion of variance explained by the fixed effect of treatment and random factor levels for species. The “ICC” (Inter-Class Correlation Coefficient) reports the proportion variance partitioned between species. The “Hot t-value” column is the t values for testing the difference between the hot treatment relative to the control and “Cold t-value” is for the cold treatment relative to the control. positive t values indicate an increase in expression and negative t values a decrease. t values > 2 and < -2 are generally significant at a p value < 0.05. The “median”, “min” and “max” columns report the median, minimum, and maximum transcripts per million (TPM) expression values across all RNA-seq libraries. For these models n = 188 and d.f. = 166. Results are ordered by the marginal R^2 (“mR2”) values.

GO ID	GO Term	Min n	mR2	cR2	ICC	Hot t-value	Hot p-value	Cold t-value	Cold p-value	median	min	max
**GO:0006457**	protein folding	41	0.32	0.53	0.21	7.48	0.0000	-3.34	0.0010	37.28	20.24	77.64
**GO:0000302**	response to reactive oxygen species	11	0.29	0.38	0.09	7.05	0.0000	-1.76	0.0810	31.16	10.32	253.50
**GO:0009408**	response to heat	37	0.29	0.39	0.10	6.25	0.0000	-2.97	0.0034	30.19	13.40	76.78
**GO:0009658**	chloroplast organization	39	0.16	0.52	0.36	4.20	0.0000	-3.47	0.0007	28.10	14.62	56.33
**GO:0010200**	response to chitin	16	0.16	0.32	0.16	-1.31	0.1908	4.93	0.0000	21.56	4.52	71.61
**GO:0018298**	protein-chromophore linkage	14	0.16	0.48	0.32	0.47	0.6416	-6.29	0.0000	76.82	20.22	263.04
**GO:0006397**	mRNA processing	72	0.15	0.42	0.27	4.67	0.0000	-2.05	0.0417	26.71	15.74	38.12
**GO:0006281**	DNA repair	34	0.13	0.43	0.30	3.47	0.0007	-2.92	0.0040	21.40	13.56	31.90
**GO:0015995**	chlorophyll biosynthetic process	13	0.13	0.52	0.39	3.61	0.0004	-3.43	0.0008	44.88	15.23	106.27
**GO:0006364**	rRNA processing	40	0.12	0.52	0.40	6.04	0.0000	0.36	0.7171	31.84	17.29	57.41
**GO:0009416**	response to light stimulus	37	0.12	0.59	0.47	2.64	0.0090	-4.64	0.0000	42.31	18.30	87.54

**GO:0006310**	DNA recombination	10	0.11	0.47	0.36	3.04	0.0028	-3.11	0.0022	18.79	8.17	36.94
**GO:0006412**	translation	70	0.11	0.56	0.45	5.73	0.0000	-0.26	0.7979	72.38	28.85	185.26
**GO:0006260**	DNA replication	11	0.10	0.63	0.53	-0.97	0.3320	-6.55	0.0000	28.51	16.07	48.66
**GO:0000398**	mRNA splicing, via spliceosome	47	0.09	0.51	0.42	2.40	0.0173	-3.28	0.0013	39.30	20.37	56.99
**GO:0007049**	cell cycle	29	0.09	0.44	0.35	1.08	0.2806	-4.13	0.0001	23.97	15.38	35.18
**GO:0009873**	ethylene-activated signaling pathway	26	0.09	0.37	0.28	-0.51	0.6083	4.20	0.0000	23.98	11.77	51.48
**GO:0051085**	chaperone cofactor-dependent protein refolding	10	0.09	0.37	0.28	2.30	0.0227	-2.70	0.0077	37.57	13.12	145.63
**GO:0008380**	RNA splicing	49	0.08	0.49	0.41	2.58	0.0107	-2.89	0.0044	30.02	17.72	46.66
**GO:0009736**	cytokinin-activated signaling pathway	10	0.08	0.38	0.30	2.18	0.0309	-2.73	0.0070	25.08	11.04	38.67

Table S2. Model results for average expression levels of orthogroups. The most common GO term (GO_ID and GO_term columns) of orthogroups with the strongest variation between treatments, indicates that the trends are similar to the GO term analysis. Full column descriptors in legend for Table S1. Results are ordered by the marginal R^2 ("mR2") values.

Orthogroup	GO_ID	GO_Term	mR2	cR2	ICC	Hot t-value	Hot df	Hot p-value	Cold t-value	Cold df	Cold p-value	median
**OG0000242**	GO:0007165	signal transduction	0.20	0.59	0.39	-0.22	102	0.8249	6.18	102	0.0000	53.02
**OG0000018**	GO:0009408	response to heat	0.15	0.37	0.22	4.81	120	0.0000	0.02	121	0.9855	29.06
**OG0000033**	GO:0007275	multicellular organism development	0.12	0.31	0.19	2.34	122	0.0208	-2.45	121	0.0157	13.55
**OG0000039**	GO:0009414	response to water deprivation	0.10	0.57	0.47	3.76	129	0.0003	-1.82	129	0.0714	105.19
**OG0000183**	GO:0002091	negative regulation of receptor internalization	0.10	0.53	0.43	-0.61	102	0.5428	3.82	102	0.0002	24.74

**OG0000285**	NA	NA	0.09	0.49	0.40	-0.64	111	0.5212	3.70	111	0.0003	81.31
**OG0000068**	GO:0006556	S-adenosylmethionine biosynthetic process	0.08	0.65	0.57	-5.29	154	0.0000	0.26	154	0.7962	137.68
**OG0000020**	GO:0007165	signal transduction	0.07	0.67	0.60	1.14	109	0.2551	4.92	109	0.0000	82.60
**OG0000435**	GO:0042823	pyridoxal phosphate biosynthetic process	0.07	0.35	0.28	-0.11	100	0.9094	-3.01	100	0.0034	148.67
**OG0000345**	GO:0009103	lipopolysaccharide biosynthetic process	0.06	0.35	0.29	-2.64	88	0.0099	0.10	87	0.9220	23.66
**OG0000047**	GO:0055085	transmembrane transport	0.05	0.31	0.26	-2.03	134	0.0440	1.26	134	0.2095	36.48
**OG0000061**	GO:0006457	protein folding	0.05	0.46	0.41	-3.23	107	0.0016	-2.57	107	0.0115	372.76
**OG0000148**	GO:0002181	cytoplasmic translation	0.05	0.51	0.46	4.13	145	0.0001	1.33	145	0.1844	48.51
**OG0000224**	GO:0071555	cell wall organization	0.05	0.64	0.59	-0.35	119	0.7233	3.65	119	0.0004	28.74
**OG0000399**	GO:0006886	intracellular protein transport	0.05	0.11	0.06	0.15	96	0.8797	2.16	96	0.0330	16.45
**OG0000015**	GO:0006986	response to unfolded protein	0.04	0.60	0.56	-3.65	141	0.0004	-1.02	141	0.3101	156.56
**OG0000054**	GO:0042026	protein refolding	0.04	0.59	0.55	2.86	120	0.0050	-0.68	120	0.4995	152.54
**OG0000082**	GO:0009231	riboflavin biosynthetic process	0.04	0.53	0.49	3.03	125	0.0030	2.85	124	0.0052	29.37
**OG0000093**	GO:0006412	translation	0.04	0.60	0.56	3.53	133	0.0006	0.47	133	0.6364	118.82
**OG0000274**	GO:0006412	translation	0.04	0.61	0.57	3.35	95	0.0012	1.27	94	0.2089	93.85

Table S3. Model results for variation in responses between biomes. The boot-strapped median expression of biological processes was averaged per species within treatments to calculate fold change (FC) values. These average log_2 FC values were compared between species from different biomes. The "Alpine_t" column is the t values for the comparison between the Alpine and temperate biomes and "Arid_t" for the Arid and temperate biomes. The top 10 models with the highest R^2 values for variation between biome groups are reported for hot and cold treatments.

GO_ID	GO_Term	min_n	treatment	R-sqrd	Alpine_t	Arid_t	p-value
GO:0009753	response to jasmonic acid	14	hot	0.40	-0.47	0.02	0.0139
GO:0000302	response to reactive oxygen species	11	hot	0.33	0.04	0.03	0.0335
GO:0035556	intracellular signal transduction	31	hot	0.32	-1.15	0.13	0.0365
GO:0006749	glutathione metabolic process	14	hot	0.30	2.68	0.27	0.0480
GO:0007165	signal transduction	30	hot	0.30	-1.33	0.22	0.0474
GO:0009611	response to wounding	36	hot	0.30	-0.39	0.06	0.0489
GO:0006412	translation	70	hot	0.29	-1.02	0.02	0.0575
GO:0006468	protein phosphorylation	50	hot	0.29	-1.04	0.16	0.0559
GO:0009408	response to heat	37	hot	0.29	1.31	0.25	0.0566
GO:0009742	brassinosteroid mediated signaling pathway	11	hot	0.29	-0.45	0.07	0.0534
GO:0034605	cellular response to heat	10	hot	0.29	1.10	0.17	0.0533
GO:0051085	chaperone cofactor-dependent protein refolding	10	hot	0.27	1.25	0.27	0.0725
GO:0006351	transcription, DNA-templated	18	cold	0.31	-1.96	0.56	0.0421
GO:0002181	cytoplasmic translation	11	cold	0.26	-2.46	0.23	0.0744
GO:0009416	response to light stimulus	37	cold	0.26	-1.35	0.33	0.0743
GO:0009740	gibberellic acid mediated signaling pathway	11	cold	0.24	-0.82	0.19	0.1016
GO:0009736	cytokinin-activated signaling pathway	10	cold	0.23	-0.51	0.13	0.1059
GO:0045892	negative regulation of transcription, DNA-templated	38	cold	0.22	-1.53	0.61	0.1170
GO:0006310	DNA recombination	10	cold	0.21	-0.97	0.31	0.1388
GO:0009873	ethylene-activated signaling pathway	26	cold	0.19	-1.65	0.97	0.1710
GO:0006260	DNA replication	11	cold	0.17	0.78	0.32	0.1961
GO:0006412	translation	70	cold	0.16	-1.81	0.35	0.2219
GO:0045292	mRNA cis splicing, via spliceosome	11	cold	0.16	-1.73	0.67	0.2173
GO:0016032	viral process	16	cold	0.15	1.27	0.12	0.2576

Table S4. Thermal tolerance acclimation between treatments. Full column descriptors in legend for Table S1. Photosynthetic tolerance was measured across five growth cabinet blocks for each trait. This gave 5 data points per species by treatment combination to calculate acclimation or shift in tolerance between the control group and treatments. Overall heat tolerance generally increased under stress but cold tolerance did not.

trait	mR2	cR2	Hot t-value	Hot p-value	Cold t-value	Cold p-value
**Tcrit_hot**	0.09	0.48	6.70	0.0000	2.57	0.0107
**Tmax_hot**	0.08	0.39	6.13	0.0000	2.46	0.0146
**Tcrit_cold**	0.00	0.38	1.47	0.1431	0.71	0.4774
**Tmax_cold**	0.00	0.32	0.76	0.4467	-0.22	0.8292
